# Structural analysis of full-length human transmembrane protein 94 argues against its classification as a P-type Mg^2+^ ATPase

**DOI:** 10.1038/s41421-025-00806-z

**Published:** 2025-06-03

**Authors:** Yuqi Li, Ye Cong, Xinyao Lou, Weiping Li, Runhao Wang, Mingyu Gong, Jiaxian Xiao, Dandan Qian, Chuangye Yan, Deshun Gong

**Affiliations:** 1 State Key Laboratory of Medicinal Chemical Biology and College of Life Sciences, Nankai University, Tianjin, China; 2 School of Life Sciences, Tsinghua University, Beijing, China; 3 Tsinghua-Peking Joint Center for Life Sciences, Tsinghua University, Beijing, China; 4 Beijing Frontier Research Center for Biological Structure, Beijing Advanced Innovation Center for Structural Biology, Tsinghua University, Beijing, China; 5 State Key Laboratory of Membrane Biology, Tsinghua University, Beijing, China

**Keywords:** Cryoelectron microscopy, Endoplasmic reticulum

Dear Editor,

In 2018, transmembrane protein 94 (TMEM94) garnered significant attention due to the correlation observed between biallelic loss-of-function variants in TMEM94 and neurodevelopmental delay, congenital heart defects, as well as distinctive facial dysmorphism^1^. This newly described disorder was named intellectual developmental disorder with cardiac defects and dysmorphic facies (IDDCDF)^2^. Notably, all mutations identified in *TMEM94* were predicted to result in truncated proteins lacking the highly conserved C-terminal domain and exhibited a significant decrease in TMEM94 expression^1^. Recently, it has been reported that TMEM94 may also be implicated in overgrowth-intellectual disability^3^ and pancreatic adenocarcinoma^4^. Despite some advancements in the pathological aspect, the structural and functional mechanisms of TMEM94 remain enigmatic. Recently, TMEM94 was identified as the long-awaited endoplasmic reticulum (ER) Mg^2+^ ATPase and plays an indispensable role in facilitating Mg^2+^ uptake into the ER. Consequently, to reflect its newfound function, TMEM94 has been officially renamed as ER magnesium ATPase (ERMA)^5^. However, several scientists have challenged these findings through bioinformatic analysis^6^. The determination of whether TMEM94 is a distinct P-type ATPase or not necessitates further experimental investigation.

Here, we initially determined the cryo-electron microscopy (cryo-EM) structure of full-length human TMEM94 in the presence of 5 mM MgCl_2_, 10 mM NaF, and 2 mM BeSO_4_ (hereafter referred to as Condition 4) at a resolution of 2.45 Å according to the gold-standard Fourier shell correlation (FSC) 0.143 criterion (Supplementary Figs. S1–S5 and Table S1). This condition is commonly used to mimic the E2P state in the Post-Albers cycle of P-type ATPases. Similar to the actuator (A), phosphorylation (P), and nucleotide-binding (N) domains observed in the P-type ATPases, TMEM94 also possesses a cytoplasmic domain referred to as CTD1, which is analogous to the A domain and positioned between transmembrane helix 2 (M2) and M3. Additionally, TMEM94 contains two other cytoplasmic domains (CTD2 and CTD3), resembling the P and N domains, respectively, located between M4 and M5, in addition to ten transmembrane helices (Fig. 1a, b). The 2.45 Å EM map exhibits excellent main chain connectivity and side chain densities in the transmembrane domain (TMD), CTD1 and CTD2 (Supplementary Fig. S4). The EM density corresponding to the CTD3 region is predominantly absent in the 2.45 Å EM map, but can be reliably assigned when a 6 Å low-pass filter is applied (Supplementary Fig. S4c).

**Fig. 1:**
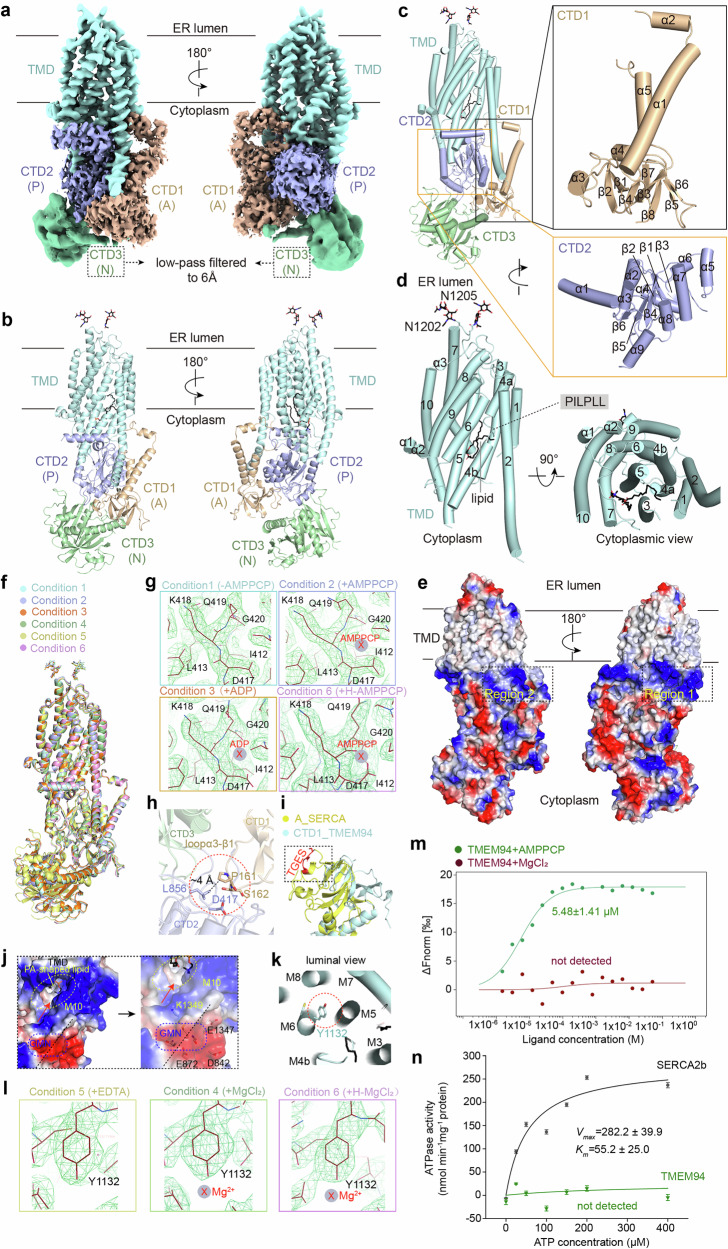
Structural analysis of human TMEM94 under six distinct conditions. **a** The overall EM density map of TMEM94. The TMD is shown in pale cyan, CTD1 in wheat, CTD2 in light blue, and CTD3 in lime. The map corresponding to CTD3 has been subjected to low-pass filtering at a resolution of 6 Å. CTD1, CTD2, and CTD3 exhibit structural similarities with the A, P, and N domains observed in P-type ATPases, respectively. **b** Overall structure of TMEM94. The *N*-linked glycans are displayed as black sticks. **c** The structural features of the CTD1 and CTD2. The CTD1 contains eight β-sheets and five α-helices. The CTD2 contains six β-sheets and nine α-helices, and the β-sheets are centrally located, surrounded by the α-helices. **d** The TMD consists of ten helices, with a discontinuity observed in M4. A lipid resembling PA is embedded within a gorge formed by M3, M4, M5, and M7. Two glycosylation sites have been identified. **e** The electrostatic potential surface of TMEM94. An intensely positively charged cytoplasmic band adjacent to the TMD, which can be divided into two regions. **f** The six structures of TMEM94 are almost identical. **g** No nucleotides were detected in the structures obtained under various nucleotide conditions. The structure of Condition 1 without the addition of nucleotide serves as a control. H, higher concentration. **h** Residues P161 and L856 introduce a steric hindrance between the D417 and CTD3. **i** The CTD1 of TMEM94 lacks the dephosphorylation motif TGES. **j** The pathway extending from the GMN motif to the lipid entry site is enriched with positively charged residues. **k** Shown here is the Mg^2+^-binding site identified previously, with Y1132 being recognized as the pivotal residue for contact. **l** No Mg^2+^ was detected in the structures obtained with varying concentrations of Mg^2+^. The structure of Condition 5 lacking Mg^2+^ was utilized as a control. **m** The dose-response curves of MST assays revealed that TMEM94 exhibits micromolar affinity for AMPPCP, whereas no interactions were detected with MgCl_2_. **n** No ATPase activity was detected for TMEM94, whereas SERCA2b exhibits obvious ATPase activity. Each data point is the average of three independent experiments, and error bars represent SD.

The CTD1 encompasses residues 15–61 and 142–265, which are situated between M2 and M3. This domain adopts a folding pattern consisting of eight β-strands and five α-helices. It is positioned in close proximity to both CTD2 and CTD3, thereby forming a tightly interconnected structure (Fig. 1c). The residues 218–239 are invisible in the map, indicating their intrinsic flexibility. The CTD2, comprising residues 374–426 and 831–1071, is positioned between M4 and M5 and adopts a folding pattern with nine α-helices and six β-strands. Notably, the β-strands are centrally located, surrounded by the nine α-helices (Fig. 1c). The CTD3, composed of residues 427–830, is positioned just below the CTD1 and CTD2, and exhibits a lower resolution reflecting its intrinsic flexibility (Fig. 1a).

The TMD, encompassing residues 62–141, 266–350, and 1072–1356, adopts a tertiary structure comprising ten transmembrane helices, including three elongated helices: M2, M5, and M10 (Fig. 1d). Similar to the P-type ATPase, M4 of TMEM94 also contains a PILPLL motif that disrupts its structure into two short helices, resembling the canonical binding site (CBS) generated by helix-breaking proline residues observed in P-type ATPases^7^. The CBS element in P-type ATPases is utilized for ligand binding, as it facilitates the formation of a spacious cavity and provides a suitable platform for the ligand to reside^7^. Notably, a phosphatidic acid (PA)-shaped lipid is embedded within a gorge formed by M3, M4, M5, and M7, with one fatty acid chain precisely adjacent to the putative CBS (Fig. 1d; Supplementary Figs. S4e, S6a, b).

Considering the recent classification of TMEM94 as a Mg^2+^-ATPase^5^, the presence of an intensely positively charged cytoplasmic band adjacent to the TMD on the electrostatic potential surface of TMEM94 is unexpected (Fig. 1e). The positively charged band can be divided into two distinct regions: region 1 comprises the cytoplasmic extension of M7, the loop between M6 and M7 (hereafter referred to as loopM6–M7), loopM8–M9, and M10; while region 2 is formed by the cytoplasmic extension of M2, α1 and α2 of CTD1, and α1 of CTD2 (Supplementary Fig. S7). Notably, a multitude of positively charged residues within region 1 form two interconnected cytosolic vestibules beside M10, which may facilitate anion selection (Supplementary Fig. S7a). Consequently, it is reasonable to hypothesize that the negatively charged PA-shaped lipid can be attracted and translocate into the TMD through these entrances (Fig. 1d; Supplementary Fig. S6a, b). Similarly, numerous positively charged residues located in region 2 also create an extensive cytosolic vestibule beside M1 and M2 (Supplementary Fig. S7b), resembling the ion entry sites in P-type ATPases^6^. Collectively, these features exhibit a preference for entry of anion rather than cation, in contrast to the recent identification of TMEM94 as a Mg^2+^ transporter.

Structural comparison revealed that the E2~P state of secretory pathway Ca^2+^ ATPase (SPCA) is the most structurally similar to TMEM94 among known P-type ATPases, exhibiting a main-chain root mean square deviation (RMSD) of ~6.9 Å for the intact structure (Supplementary Fig. S8). To further experimentally investigate whether TMEM94 functions as a Mg^2+^-ATPase, we employed cryo-EM techniques to determine the structures of TMEM94 under five additional distinct conditions, which are commonly employed to mimic intermediate states within the well-established Post-Albers catalytic cycle of P-type ATPases (Supplementary Fig. S9a). Specifically, these conditions include: Condition 1 mimicking the MgE1 state; Condition 2 mimicking the MgE1-ATP state; Condition 6 mimicking the MgE1-ATP(H) state by using elevated concentrations of MgCl_2_ and AMPPCP to account for potentially weak binding affinities of TMEM94 to Mg^2+^ and AMPPCP; Condition 3 mimicking the MgE1P-ADP state; and Condition 5 mimicking the E2P state. Five structures were determined at overall resolutions of 2.59 Å (Condition 1), 2.49 Å (Condition 2), 3.24 Å (Condition 3), 2.82 Å (Condition 5), and 2.92 Å (Condition 6), respectively (Supplementary Figs. S3, S9). These high-resolution structures enable precise differentiation between ion- and nucleotide-binding sites, as well as detailed characterization of conformational changes.

Notably, all six structures exhibit a nearly identical conformation, with the mean RMSD value relative to the Condition 5 structure being ~0.49 Å (Fig. 1f). When comparing the structure of Condition 1 in the absence of nucleotides with the other three structures in the presence of varying concentrations of nucleotides (ADP or AMPPCP), we did not observe nucleotides surrounding the previously proposed phosphorylation site D417^5^, even at a higher AMPPCP concentration (Fig. 1g). However, the dose-response curve obtained from the microscale thermophoresis (MST) assay reveals that TMEM94 exhibits micromolar affinity for AMPPCP (Fig. 1m), suggesting that the invisible flexible region of TMEM94 might play a critical role in ATP binding. Interestingly, the AlphaFold3-predicted structure of the TMEM94–ATP-Mg^2+^ complex reveals that ATP-Mg^2+^ is positioned within the flexible region of TMEM94 (Supplementary Fig. S10a). In contrast, in the predicted structure of the plasma membrane Ca^2+^ ATPase 1 (PMCA1)–ATP-Mg^2+^ complex, ATP-Mg^2+^ resides at the canonical nucleotide-binding site of P-type ATPases (Supplementary Fig. S10b), although the detailed structural features of this complex remain unresolved. Furthermore, the residue P161 located in the loopα3–β1 of CTD1 and residue L856 in CTD2 may introduce steric hindrance that impedes phosphorylation of D417 (Fig. 1h). Comparison of the CTD1 with the A domain of sarco/endoplasmic reticulum calcium ATPase (SERCA) reveals the absence of the crucial dephosphorylation motif TGES in CTD1 (Fig. 1i). These observations suggest that TMEM94 does not undergo phosphorylation-dephosphorylation reactions in our structural analysis.

In a previous study, a GMN motif and the Y1132 residue (Fig. 1k) are identified as two sites for Mg^2+^ binding^5^. In our structure, the GMN motif is positioned at the cytoplasmic terminus of M10, distanced from the positively charged cytoplasmic vestibule responsible for facilitating lipid entry (Fig. 1j; Supplementary Fig. S6c). Despite the presence of a region rich in negatively charged residues adjacent to the GMN motif, the ion entry pathway into the TMD is positively charged (Fig. 1j), thereby precluding Mg^2+^ entry. Furthermore, even under extremely high Mg^2+^ conditions, no discernible density corresponding to Mg^2+^ was observed in the vicinity of the GMN motif, the Y1132 residue (Fig. 1k, l), or any other resolved regions of TMEM94. Notably, the MST binding assay indicates that no interaction between TMEM94 and Mg^2+^ was detected (Fig. 1m). These observations suggest that TMEM94 does not bind to Mg^2+^ under detergent micelle conditions. In addition, no detectable ATPase activity was observed for TMEM94, in contrast to the positive control SERCA2b, which exhibited obvious ATPase activity. The *K*_m_ and *V*_max_ values for the ATPase activity of SERCA2b were determined to be ~55.2 µM and 282.2 nmol·min^−1^·mg^−1^, respectively (Fig. 1n; Supplementary Fig. S11a). Moreover, we mapped six pathogenic mutations onto the TMEM94 structure. All of them could be reliably located and exhibit varying degrees of cytoplasmic domain deficiency (Supplementary Fig. S11b), thereby reflecting its physiological importance.

In this study, we report six high-resolution structures of human TMEM94 at resolutions ranging from 2.45 Å to 3.24 Å. These structures reveal the architectural details of the TMEM94 family and provide evidence for the absence of key features, such as ATP binding at the proposed phosphorylation site D417, Mg^2+^ binding, phosphorylation-dephosphorylation reactions, and the Post-Albers catalytic cycle in TMEM94 under detergent micelle conditions. However, we cannot rule out the possibility that TMEM94 undergoes autoinhibition under detergent micelle conditions or that essential factors may be missing during protein purification. Therefore, whether TMEM94 can function as a P-type Mg^2+^ ATPase remains to be further validated. Additionally, the interaction between TMEM94 and PA-shaped lipid suggests that lipids might modulate TMEM94 activity or that TMEM94 might be involved in lipid transport; however, the physiological significance of this interaction remains to be elucidated.

## Supplementary information

Supplementary Information

## Data Availability

The atomic coordinates and electron microscopy density maps of the six structures have been deposited in the Protein Data Bank (PDB, http://www.rcsb.org) and the Electron Microscopy Data Bank (EMDB, https://www.ebi.ac.uk/pdbe/emdb/). The accession numbers are as follows: Condition 1 (PDB: 9JJK; EMDB: EMD-61530); Condition 2 (PDB: 9JJN; EMDB: EMD-61533); Condition 3 (PDB: 9JJO; EMDB: EMD-61534); Condition 4 (PDB: 9JK3; EMDB: EMD-61542); Condition 5 (PDB: 9JK4; EMDB: EMD-61543); Condition 6 (PDB: 9JK5; EMDB: EMD-61544).

## References

[1] Stephen, J. et al. *Am. J. Hum. Genet*. **103**, 948–967 (2018). 10.1016/j.ajhg.2018.11.001 PMC6288279 30526868

[2] Al-Hamed, M. H. et al. *Genes* **11**, 967 (2020). 32825426 10.3390/genes11090967 PMC7565137

[3] Zhou, Y., Huang, B., Zhang, Q., Yu, Y. & Xiao, J. *Transl. Cancer Res.* **13**, 1425–1442 (2024). 38617519 10.21037/tcr-23-1365 PMC11009810

[4] Yuksel Ulker, A. et al. *Am. J. Med. Genet. A* **191**, 1530–1545 (2023). 10.1002/ajmg.a.63180 36919607

[5] Vishnu, N. et al. *Mol. Cell* **84**, 1321–1337 (2024). 38513662 10.1016/j.molcel.2024.02.033 PMC10997467

[6] Palmgren, M., Morth, J. P. & Nissen, P. *Cell Calcium* **123**, 102911 (2024). 38879951 10.1016/j.ceca.2024.102911

[7] Palmgren, M. *J. Biol. Chem.* **299**, 105352 (2023). 37838176 10.1016/j.jbc.2023.105352 PMC10654040

