# Supplementary material for: Structural analysis of full-length human transmembrane protein 94 argues against its classification as a P-type Mg2+ ATPase
Source: Cell Discov. 2025 Jun 3;11:54. doi: 10.1038/s41421-025-00806-z (PMC12134183; doi:10.1038/s41421-025-00806-z)
Supplement: Supplementary file 1 — Supplementary Information [file 41421_2025_806_MOESM1_ESM.pdf]

Supplementary Materials for

Structural analysis of full-length human transmembrane protein 94 argues against its classification as a P-type Mg^{2+} ATPase

Yuqi Li[1#], Ye Cong[2#], Xinyao Lou[2], Weiping Li[1], Runhao Wang[1], Mingyu Gong[1], Jiaxian Xiao[1], Dandan Qian[1*], Chuangye Yan[2*], Deshun Gong[1*]

*Correspondence to:

qiandd@nankai.edu.cn; yancy2019@tsinghua.edu.cn; gongds@nankai.edu.cn.

This PDF file includes:

Materials and Methods

Supplementary Figs. S1-S11

Supplementary Table S1

References 1-11

Materials and Methods

Transient protein expression and purification

The full-length human *TMEM94* cDNA was generated from HEK293F cell (Invitrogen)-derived RNA and subsequently subcloned into the pCAG vector, with incorporation of a C-terminal FLAG-tag and His_{10}-tag. The HEK293F cells were cultured in SMM 293T-II medium (Sino Biological Inc.) at 37 °C under a 5% CO_2 atmosphere using a Zhichu shaking incubator (ZQWY-AS8E, 120 rpm). Upon reaching a cell density of 2.0×10^6 cells per ml, the pCAG-*TMEM94* plasmids were transiently transfected into the cells. For a 1-liter HEK293F cell culture, approximately 1.5 mg of plasmids were pre-incubated with 4.0 mg of linear polyethylenimines (PEIs) (Polysciences) with a molecular weight of 25-kDa in 50 ml fresh medium for 20-30 minutes prior to transfection. The resulting mixture was then added to the cell culture and incubated for 48 hours before harvesting.

For purification, HEK293F cells (5 liters) were harvested by centrifugation at 800g for 10 minutes and resuspended in lysis buffer A, which contained 25 mM Tris (pH 8.0), 150 mM NaCl. Additionally, the lysis buffer A was supplemented with aprotinin (1.3 μg/ml), pepstatin (1 μg/ml), leupeptin (5 μg/ml), and 5 mM $MgCl_2$. The lysate was then incubated with a mixture of n-dodecyl-β-D-maltopyranoside (DDM) at a concentration of 1.5 % and cholesteryl hemisuccinate tris salt (CHS) at a concentration of 0.15 % for membrane protein extraction at 4 °C for two hours. After ultracentrifugation at 18,700g for 1 hour, the supernatant was collected and applied to the Anti-DYKDDDDK Tag

(L5) Affinity Gel (BioLegend) at 4 °C for two rounds. The resin was washed six times with 5 ml of wash buffer A (lysis buffer A supplemented with 0.006 % glycol-diosgenin (GDN, Anatrace) and 5 mM $MgCl_2$) each time. The protein was eluted using elution buffer A (wash buffer A containing 200 μg/ml FLAG peptide (GL Biochem)). Subsequently, the eluent mixed with 10 mM imidazole (pH 8.0) was incubated with nickel affinity resin (Ni-NTA, Qiagen) for a duration of 2 hours at 4 °C. The resin was then washed using wash buffer B (lysis buffer A supplemented with 0.006% GDN and 30 mM imidazole and 5 mM $MgCl_2$), followed by protein elution using elution buffer B (lysis buffer A supplemented with 0.006% GDN and 300 mM imidazole and 5 mM $MgCl_2$). The resulting eluent was concentrated and subjected to size-exclusion chromatography (SEC) using Superose™6 Increase column(10/300, GE Healthcare) in a HEPES-based buffer containing25 mM Tris (pH8.0), 150mM NaCl, 0.006 % GDN, and 5 mM $MgCl_2$. The fractions corresponding to the peak were pooled together and further concentrated to approximately 10 mg/ml prior to cryo-electron microscopy analysis. These proteins were utilized for the preparation of Conditions 1, 2, 3, 4, and 6 samples. Significantly, 5 mM $MgCl_2$ was intentionally omitted throughout the purification process, while 5 mM EDTA was incorporated into the extraction buffer for the Condition 5 sample. For the TMEM94 proteins used in the MST binding assay, 5 mM EDTA was included in the extraction buffer, and no $MgCl_2$ was added in subsequent steps. For the human SERCA2b proteins analyzed in the MST binding assay, lysis buffer A, supplemented with 1% Lauryl maltose neopentyl glycol (LMNG) and 0.1% CHS, was utilized to extract SERCA2b proteins from membranes. Gel filtration

was performed using a buffer consisting of lysis buffer A plus 0.006% GDN. The purification protocol for SERCA2b is identical to that of TMEM94.

Preparation of Cryo-EM samples

The metal fluoride compounds, BeF_x, AlF_x, and MgF_x, function as phosphate analogues to stabilize distinct intermediate states of P-type ATPases during phosphoryl transfer/hydrolysis reactions[1]. To simulate the intermediate states within the Post-Albers catalytic cycle, TMEM94 was subjected to incubation with various substrates under the specified conditions[2]: the TMEM94 purified in the presence of 5 mM $MgCl_2$ was designated as the Condition 1. The Condition 2 was captured using 1 mM ATP analog β, γ-methyleneadenosine 5'-triphosphate (AMPPCP), while the Condition 3 was captured using 5 mM ADP, 5 mM NaF, and 1 mM $AlCl_3$. The Condition 4 state was captured using 10 mM NaF and 2 mM BeSO4. Specifically, Condition 6 state was captured using a combination of 100 mM $MgCl_2$ and 5 mM AMPPCP. To capture the Condition 5, TMEM94 purified in the presence of 5 mM EDTA was used along with10 mM NaF and 2 mM $BeSO_4$.

Cryo-EM data acquisition

The holey carbon grids (Quantifoil Au 300 mesh, R1.2/1.3) were subjected to glow-discharge in the Plasma Cleaner PDC-32G-2 (Harrick Plasma Company) under vacuum conditions for a duration of 2 minutes and at medium force for 30 seconds. Subsequently, aliquots (4 μl) of TMEM94 proteins were carefully deposited onto the

glow-discharged grids, which were then gently blotted for a period of 3 seconds and rapidly frozen by plunging into liquid ethane cooled by liquid nitrogen using Vitrobot Mark IV (Thermo Fisher Scientific), maintaining a temperature of 8°C and humidity level at 100%. The grids were loaded onto a 300 kV Titan Krios (Thermo Fisher Scientific Inc.) equipped with a K3 Summit detector (Gatan) and GIF Quantum energy filter. Images were automatically collected using AutoEMation[3] in super-resolution mode at a nominal magnification of 81,000 × (96,000 × for the Conditions 1, 2, and 6 datasets; 64,000 × for the Condition 5 dataset), with a slit width of 20 eV on the energy filter. For Conditions 1, 2, and 6 datasets, high-quality micrographs were automatically collected using EPU (Thermo Fisher Scientific Inc.) in super-resolution mode at a nominal magnification of 96,000 × on a 300 kV Titan Krios (Thermo Fisher Scientific Inc.) equipped with a Falcon4 Summit detector (Thermo Fisher Scientific Inc.). A defocus series ranging from -1.3 μm to -1.8 μm was applied. Each stack was exposed for 2.56 s with an exposure time of 0.08 s per frame, resulting in a total of 32 frames per stack and an approximate total dose of 50 e-/Å^2 for each stack. The stacks underwent motion correction using MotionCor2[4] and were binned by a factor of two, resulting in a pixel size of 0.8374 Å/pixel (0.83 Å/pixel for the Conditions 1, 2, and 6 datasets; 1.0979 Å/pixel for the Condition 5 dataset). Meanwhile, dose weighting was performed[5]. The defocus values were estimated with Gctf[6].

Image processing

The contrast transfer function (CTF) estimation in cryoSPARC[7] was performed using

dose-weighted micrographs and Patch-CTF. Micrographs with CTF fitting resolution worse than 4.0 Å were excluded during manual curation. Initial particle picking was conducted from high-quality micrographs using the blob picker tool in cryoSPARC[7], followed by generating 2D averages. Template picker was then employed for final particle picking, utilizing templates derived from the 2D results. Particles were extracted and cropped using Bin2 (binning factor of 2) parameters to expedite early-step calculations, and the resulting good particles were re-extracted for final refinement.

For Condition 4 dataset, 4,125 micrographs were collected and 5,636,637 particles were auto-picked using blob picker in cryo-SPARC. After several rounds of 2D classification, 266,095 good particles with different view directions were selected for template picking and Ab-initio reconstruction to generate initial references. Subsequently, Heterogeneous Refinement was performed on a full set of 5,636,637 particles selected by the template picker using references generated from Ab-initio reconstruction. This process resulted in the generation of a high-resolution map with a resolution of 3.41 Å and included 747,598 well-selected particles. The yielded good particles were then re-extracted using Bin1 (binning factor of 1) parameters and subjected to Heterogeneous Refinement and Non-uniform Refinement, finally generating a density map reported at an overall resolution of 2.45 Å using 580,228 particles. The data processing workflow for the remaining five datasets is analogous to that of the Condition 4 dataset. Ultimately, density maps were obtained for the Conditions 1, 2, 3, 5, and 6 datasets at resolutions of 2.45 Å, 2.49 Å, 3.24 Å, 2.82 Å,

and 2.92 Å, respectively. Resolutions were estimated using the gold-standard Fourier shell correlation (FSC) 0.143 criterion[8]. Local resolution variations were estimated in cryoSPARC.

Model building and structure refinement

The initial structure model for human TMEM94 was generated using AlphaFold[9], a state-of-the-art protein folding prediction algorithm. Subsequently, the predicted cytoplasmic domain 1 (CTD1) and CTD2 and transmembrane domain (TMD) structures were accurately positioned within the density map through molecular docking techniques. Manual adjustments and rebuilding of the CTDs and TMD structures were performed using COOT[10] software. Additionally, sequence assignment was primarily guided by bulky amino acid residues such as F, Y, W, and R to ensure accuracy. Furthermore, unique sequence patterns were exploited to validate the assigned residues. The predicted structure of CTD3 was successfully docked into a low-pass filtered map with a resolution of 6 Å, allowing for reliable orientation assignment. Structure refinements were carried out by Phenix in real space with secondary structure and geometry restraints[11]. The statistics of the 3D reconstruction and model refinement are summarized in Supplementary Table S1.

MST binding assay

The binding affinities of TMEM94 with AMPPCP or $MgCl_2$ were assessed by incubating labeled protein at a final concentration of 200 nM with AMPPCP or $MgCl_2$ at 16 different concentrations. These concentrations were obtained through a 16-step

(3n) serial dilution process, starting from 100 mM in the buffer containing 25 mM Tris pH8.0, 150 mM NaCl, 0.006% GDN, and 5 mM $MgCl_2$ ($MgCl_2$ was omitted for the $MgCl_2$ binding assay). Subsequently, the samples were loaded into Monolith MO-K022 capillaries (Nano-Temper Technologies) and incubated at room temperature for 10 min. MST studies were conducted using a Monolith NT.115 apparatus and MO. Control v1.6 software (Nano-Temper Technologies) at 25 °C with light-emitting diode (LED) power set at 20% and MST power at 40%. The Kd value was calculated using the MO. Affinity Analysis v.2.2.4 software.

ATPase activity assay

The hTMEM94 and hSERCA2b proteins used for ATPase activity assay were purified as described above. The ATPase activity was measured using QuantiChrom ATPase/GTPase assay kit (BioAssay Systems). The protein concentrations of TMEM94 and SERCA2b for the assays were 0.1 mg/ml. All reactions were performed using the reaction buffer from the assay kit with indicated ATP. For SERCA2b, the system was supplemented with 5 mM $CaCl_2$ and 5 mM $MgCl_2$; for TMEM94, the system contained 5 mM $MgCl_2$. Reactions were carried out at 37 °C for 10 min and stopped by addition of the reagent from assay kit. The mixture was incubated for 30 min at room temperature before the activity was measured by monitoring the increase of absorbance at 620 nm. Nonlinear regression to the Michaelis-Menten equation and data analysis was performed using OriginPro 8.

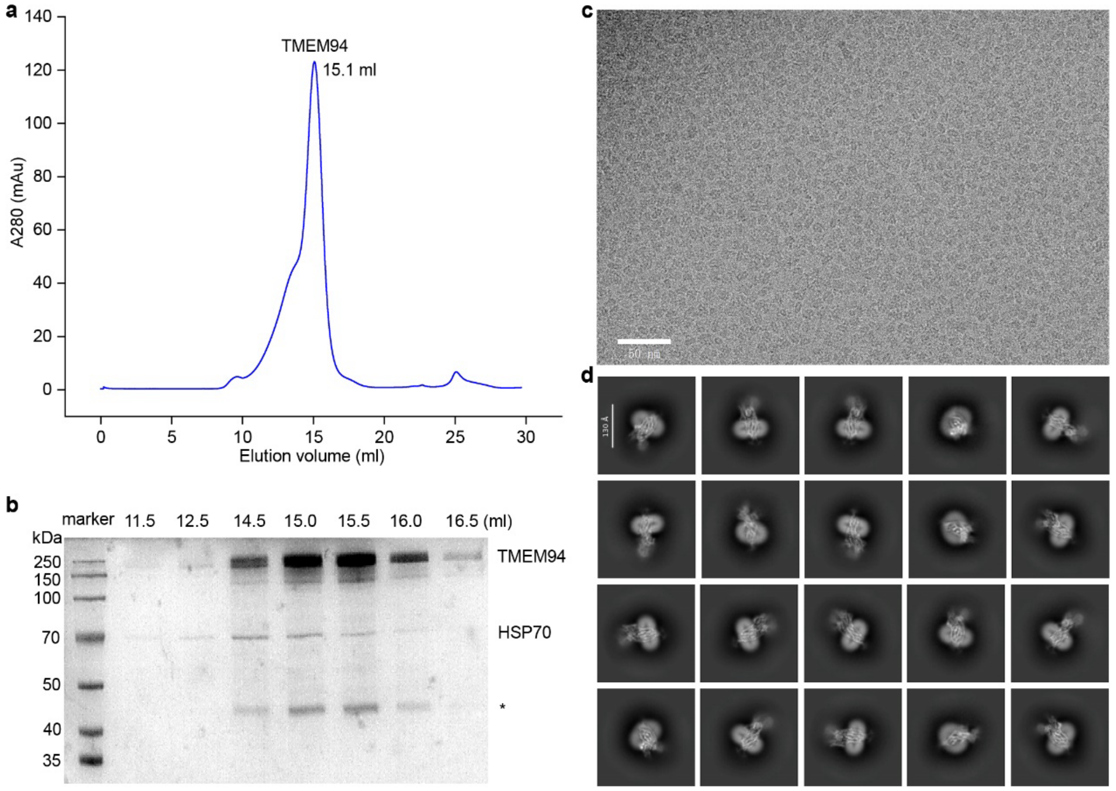

Supplementary Fig. S1 | Protein purification and cryo-EM analysis of human TMEM94. a, The TMEM94 protein was subjected to size exclusion chromatography (SEC). **b,** Peak fractions were visualized by Coomassie blue staining (down). kDa, kilodaltons. Black star signifies contamination. **c,** Representative cryo-EM micrograph of TMEM94. **d,** Representative 2D class averages of TMEM94.

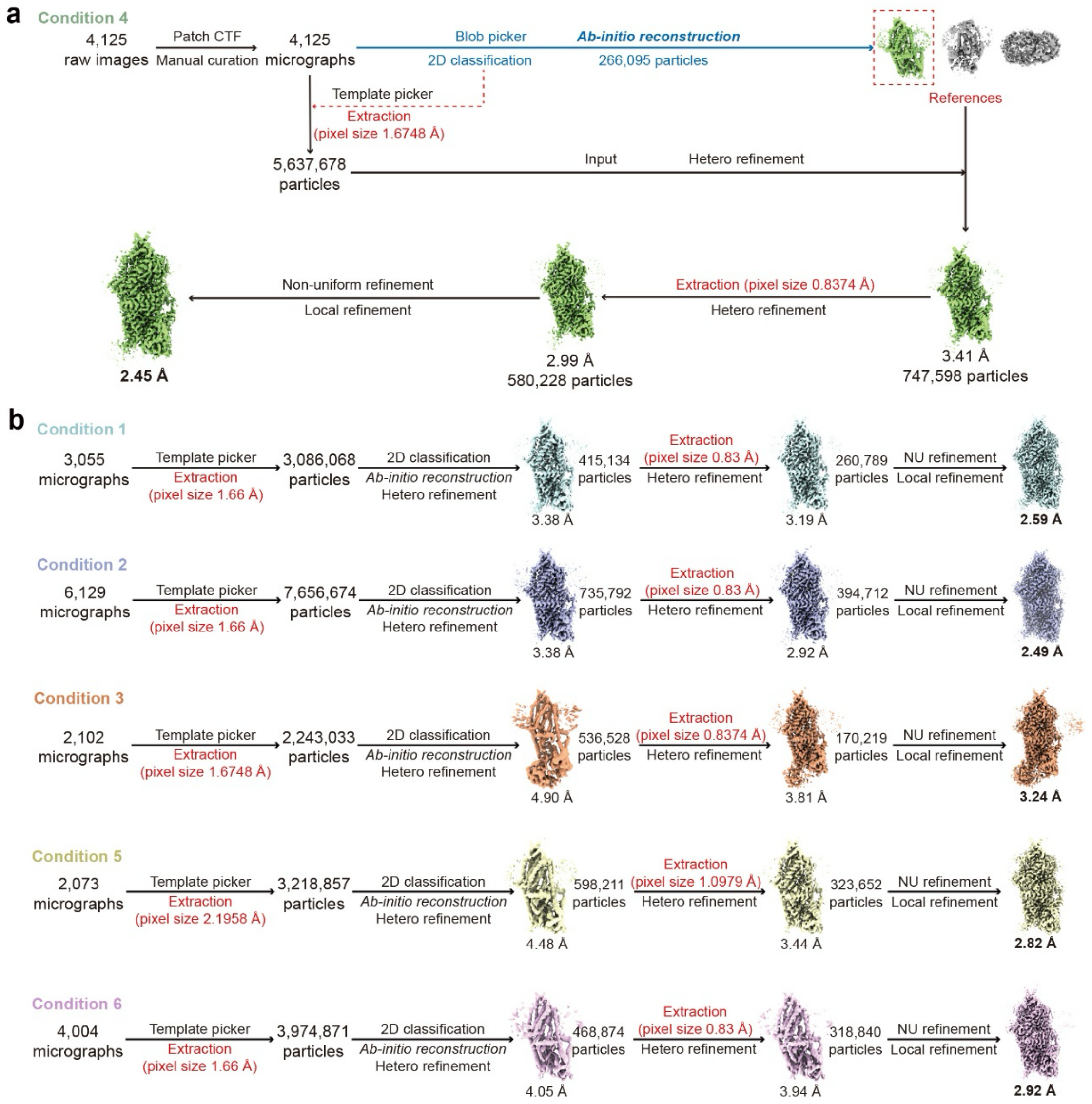

Supplementary Fig. S2 | Flowchart for cryo-EM data processing.

Please refer to Materials and Methods for details.

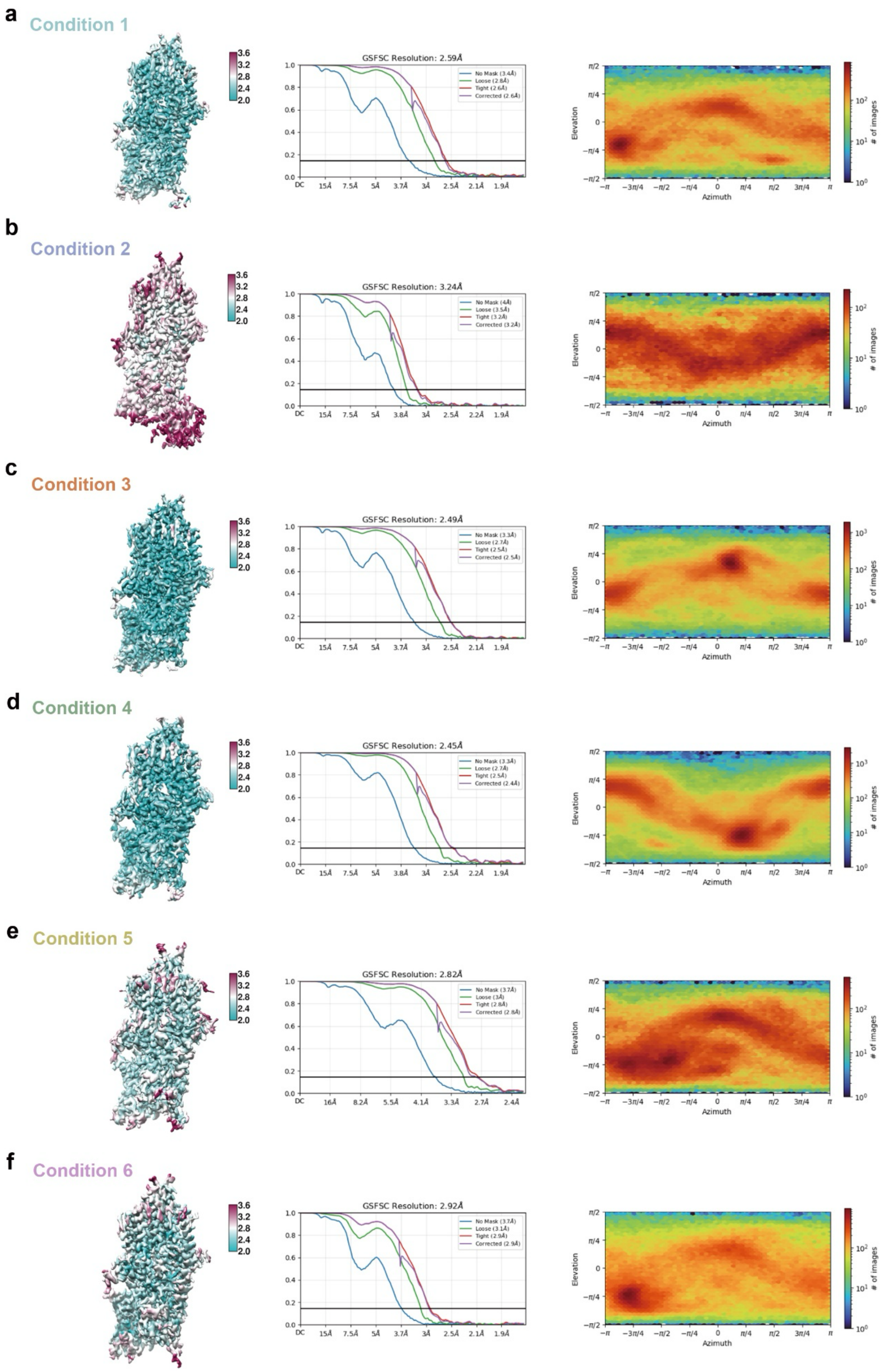

Supplementary Fig. S3 | Local resolution maps, resolution estimation, and angular

distribution of the six reconstructions. a-f, Local resolution maps, resolution estimation, and angular distribution of the six structures.

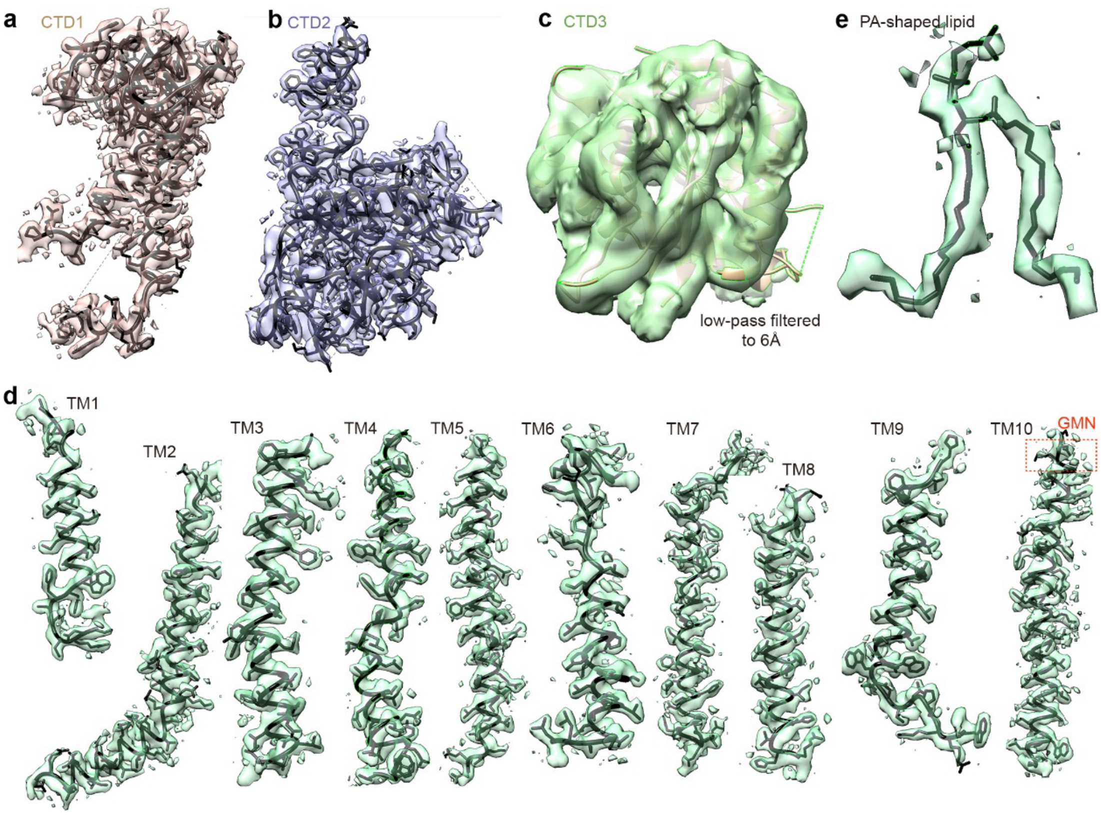

Supplementary Fig. S4 | Representative local EM maps of TMEM94. a, The EM map of the CTD1. **b,** The EM map of the CTD2. **c,** The low-pass filtered map of the CTD3. **d,** The EM maps of the ten TM helices. **e,** The EM map of the observed phosphatidic acid (PA)-shaped lipid.

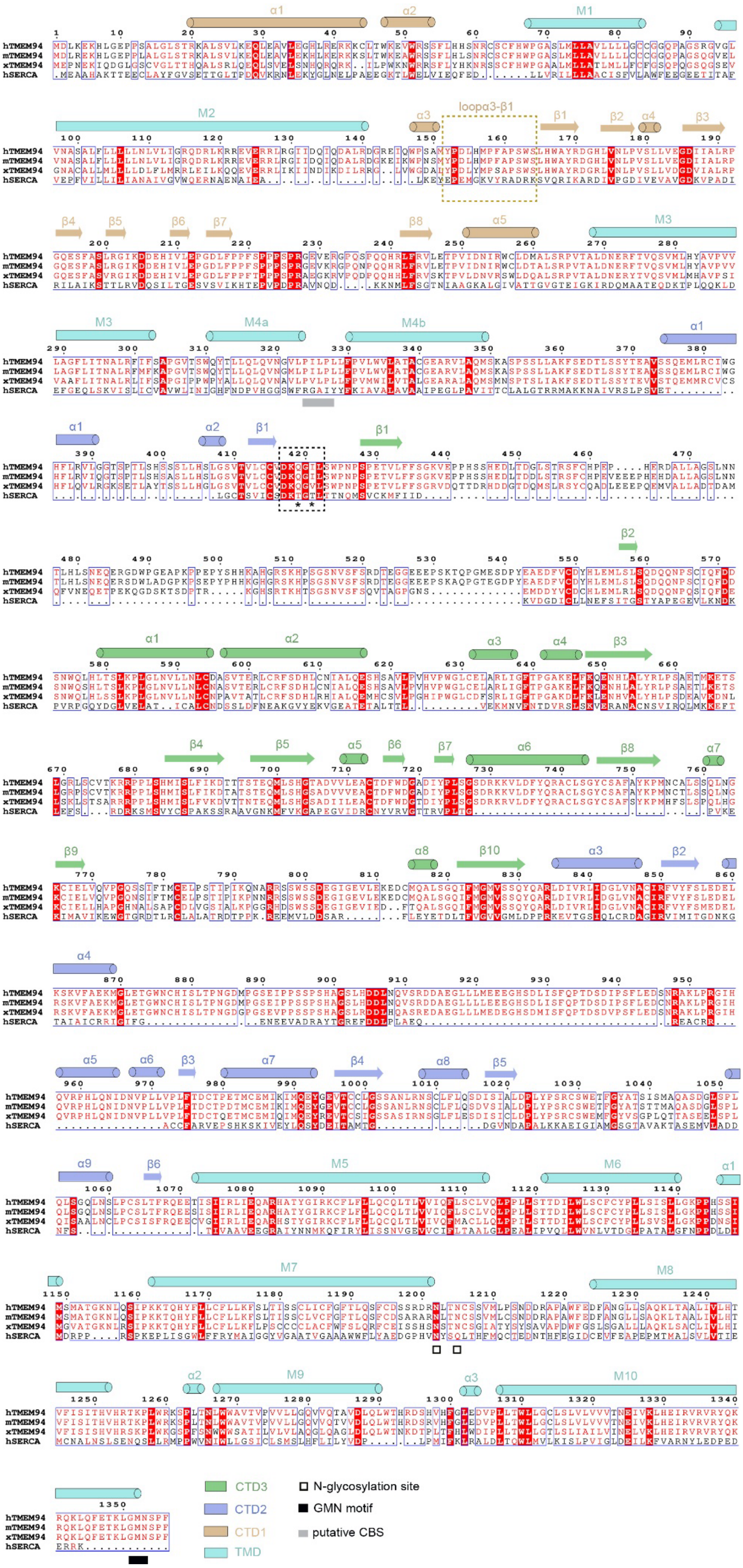
CTD3
CTD2
CTD1
TMD
N-glycosylation site
GMN motif
putative CBS

Supplementary Fig. S5 | Sequence alignment of TMEM94 with its homologues and SERCA. h, *homo sapiens*; m, *Mus musculus*; x, *Xenopus laevis.* The black dashed box highlights the DKQGIL motif, while the two black stars indicate the two key threonine residues in hSERCA. The yellow dashed box highlights the loopα3-β1.

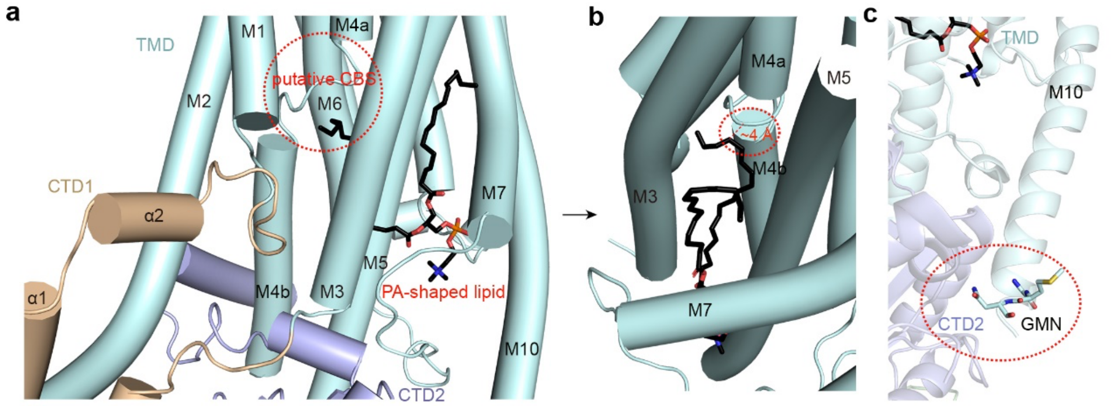

Supplementary Fig. S6 | Binding site of the observed lipid and the location of the GMN motif. a,b, Two distinct views on the binding site of the PA-shaped lipid. **c,** The GMN motif is located at the cytoplasmic terminus of M10.

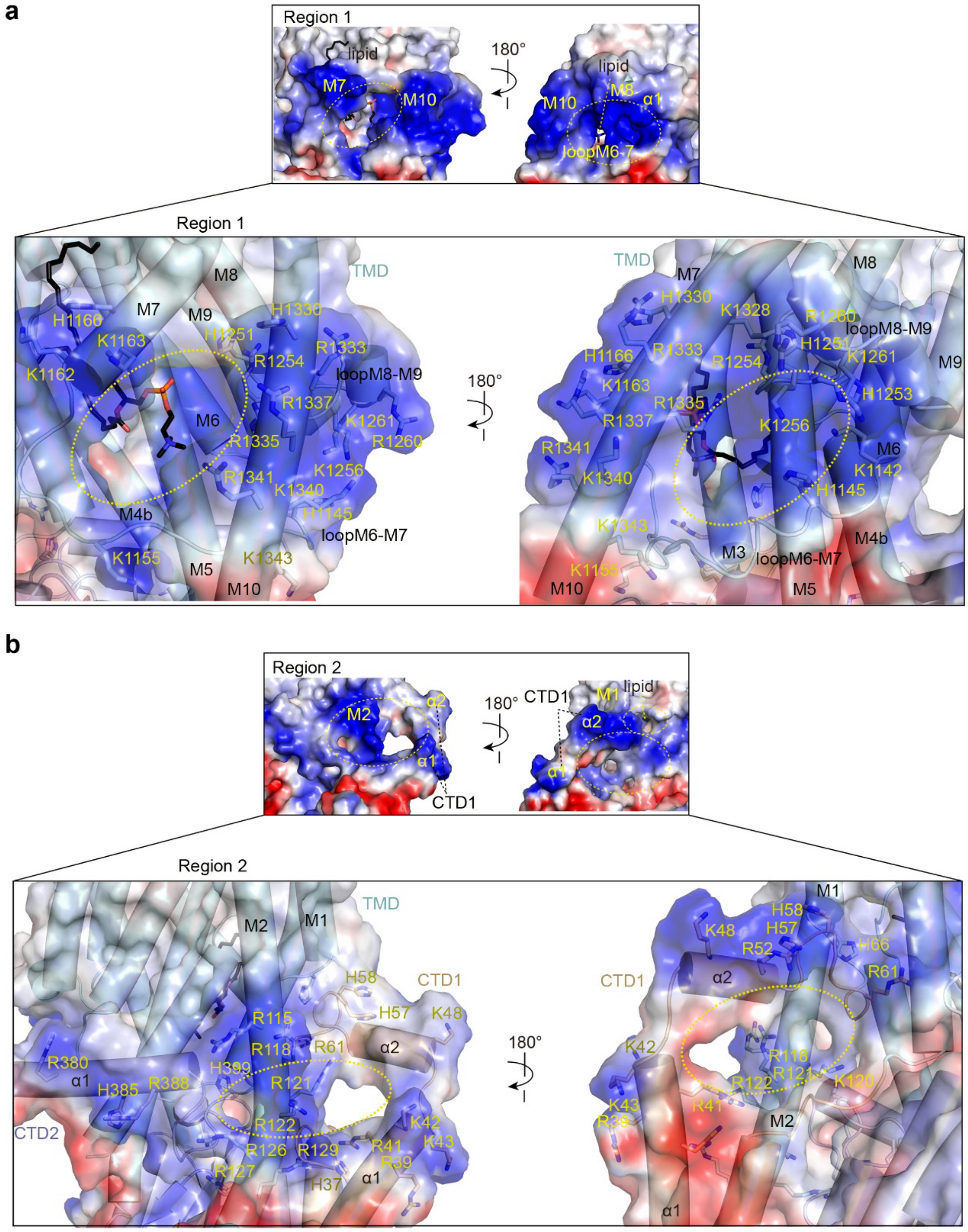

Supplementary Fig. S7 | The positively charged residues within the region 1 and 2.

a, The positively charged residues within the region 1 are indicated. **b,** The positively charged residues within the region 2 are indicated.

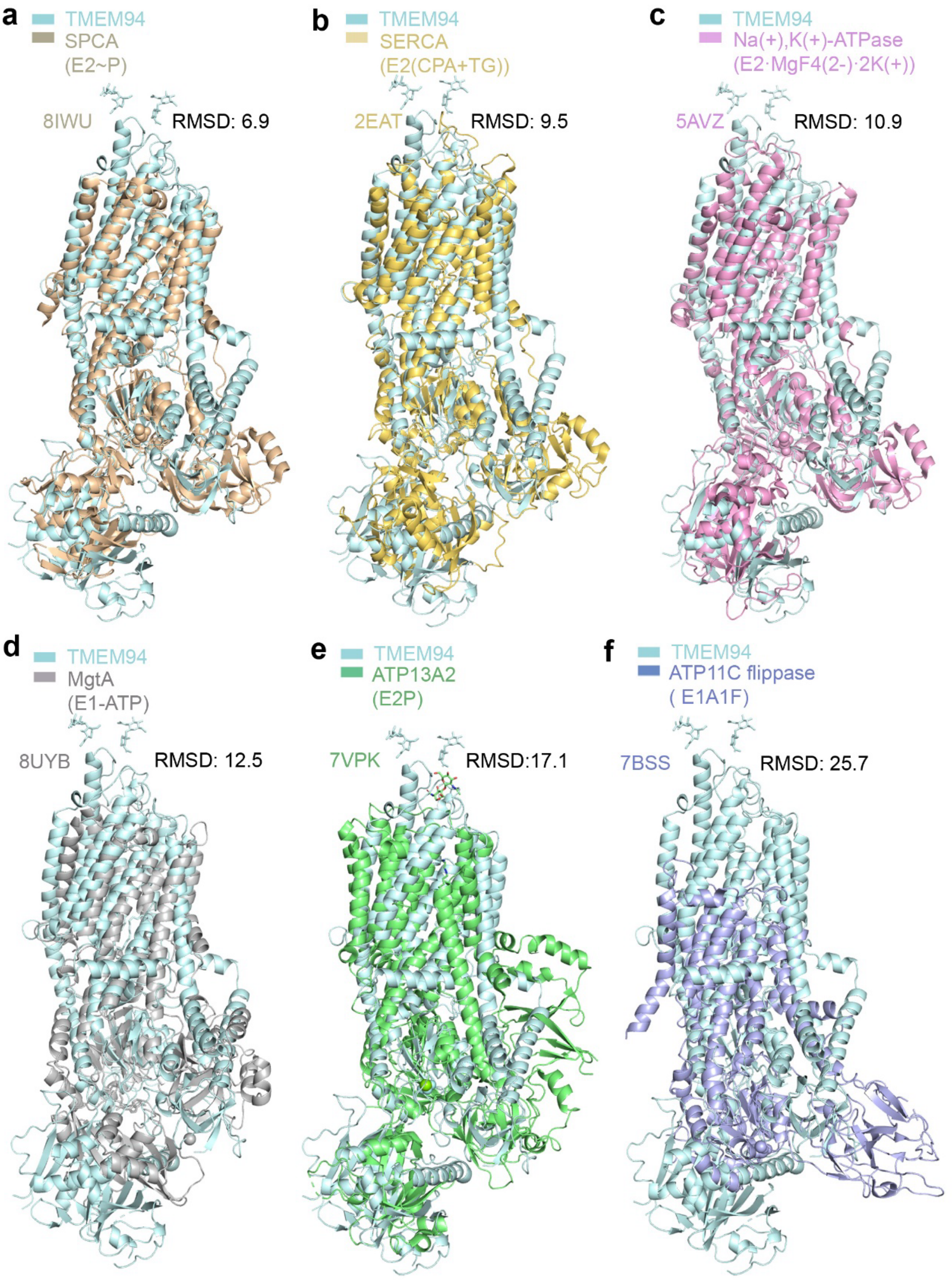

Supplementary Fig. S8 | Structure homology search of TMEM94 using DALI server. Figures a-f highlight members of the P-type ATPase family positioned at the top of the comparison list.

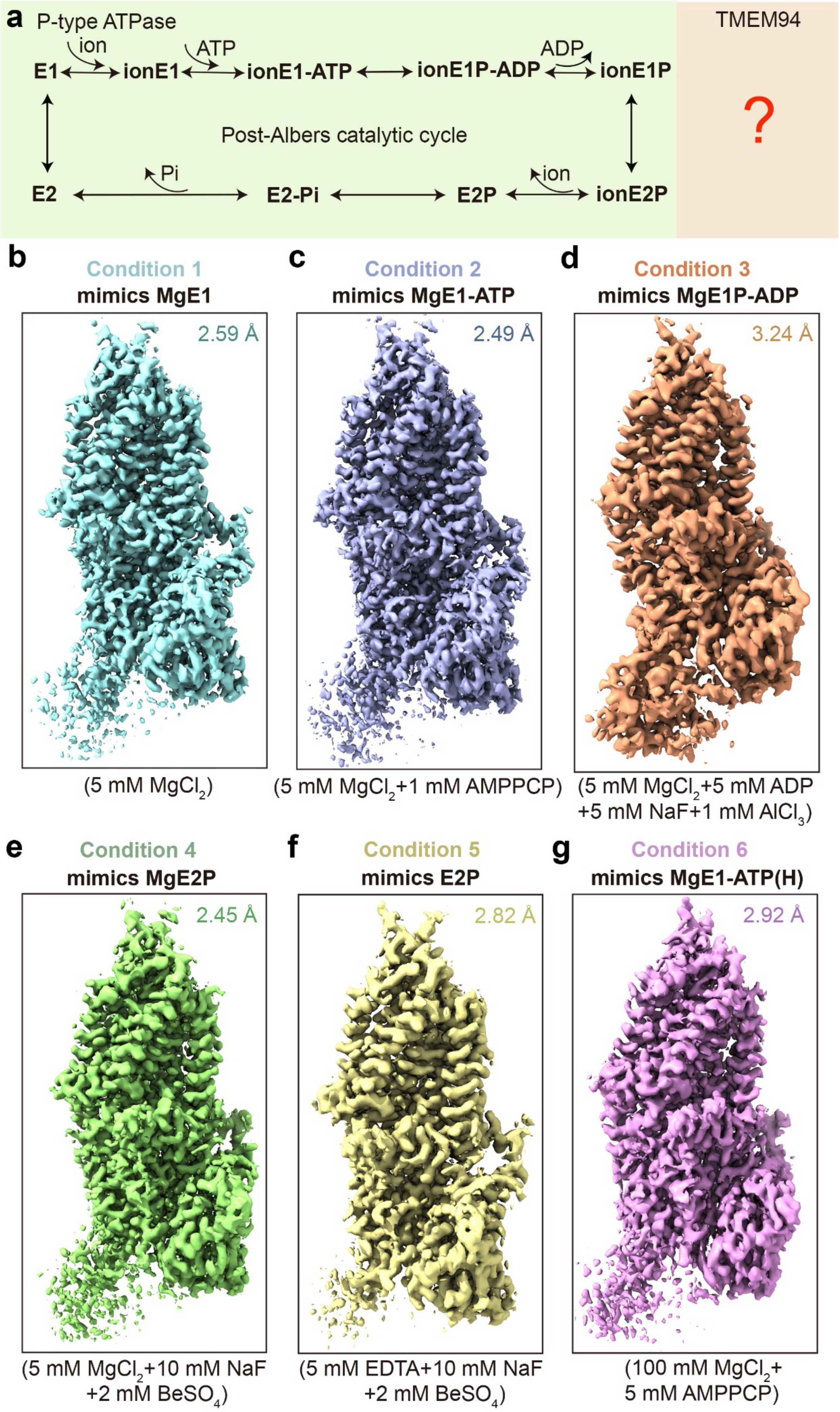
a
P-type ATPase
ion
ATP
ADP
E1
ionE1
ionE1-ATP
ionE1P-ADP
ionE1P
Post-Albers catalytic cycle
Pi
ion
E2
E2-Pi
E2P
ionE2P
TMEM94
?
b
Condition 1
mimics MgE1
2.59 Å
(5 mM MgCl2)
c
Condition 2
mimics MgE1-ATP
2.49 Å
(5 mM MgCl2+1 mM AMPPCP)
d
Condition 3
mimics MgE1P-ADP
3.24 Å
(5 mM MgCl2+5 mM ADP +5 mM NaF+1 mM AlCl3)
e
Condition 4
mimics MgE2P
2.45 Å
(5 mM MgCl2+10 mM NaF +2 mM BeSO4)
f
Condition 5
mimics E2P
2.82 Å
(5 mM EDTA+10 mM NaF +2 mM BeSO4)
g
Condition 6
mimics MgE1-ATP(H)
2.92 Å
(100 mM MgCl2+ 5 mM AMPPCP)

Supplementary Fig. S9 | Capture the putative intermediated states within the Post-Albers cycle for TMEM94. a, Post-Albers catalytic cycle observed in P-type ATPase. Does TMEM94 exhibit the Post-Albers catalytic cycle? **b-g,** The overall EM density maps for the six structures that mimic the six intermediated states in the Post-Albers catalytic cycle. The structures of the Condition 1 through 6 were determined at overall resolutions of 2.59 Å, 2.49 Å, 3.24 Å, 2.45 Å, 2.82 Å, and 2.92 Å respectively.

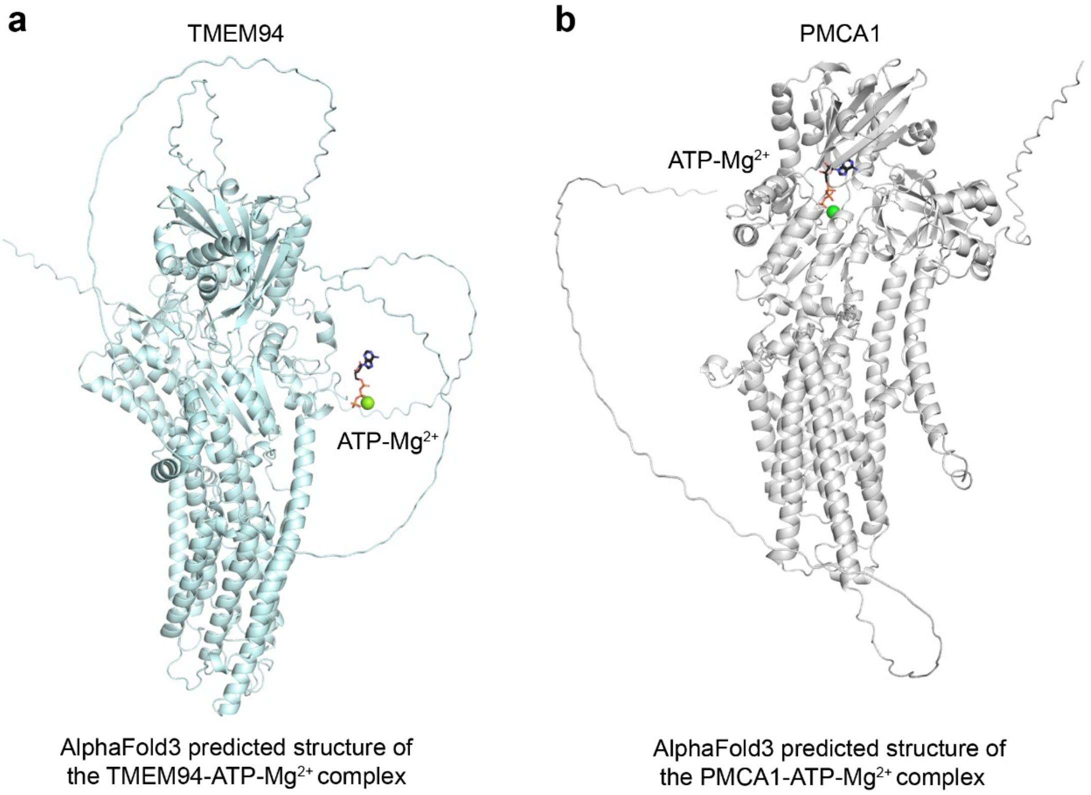

Supplementary Fig. S10 | AlphaFold3 predicted structures of TMEM94 and PMCA1 in complex with ATP-Mg^{2+}, respectively. a, For TMEM94, ATP-Mg^{2+} is positioned within the flexible region. **b,** For PMCA1, ATP-Mg^{2+} is located at the canonical nucleotide-binding site of P-type ATPases.

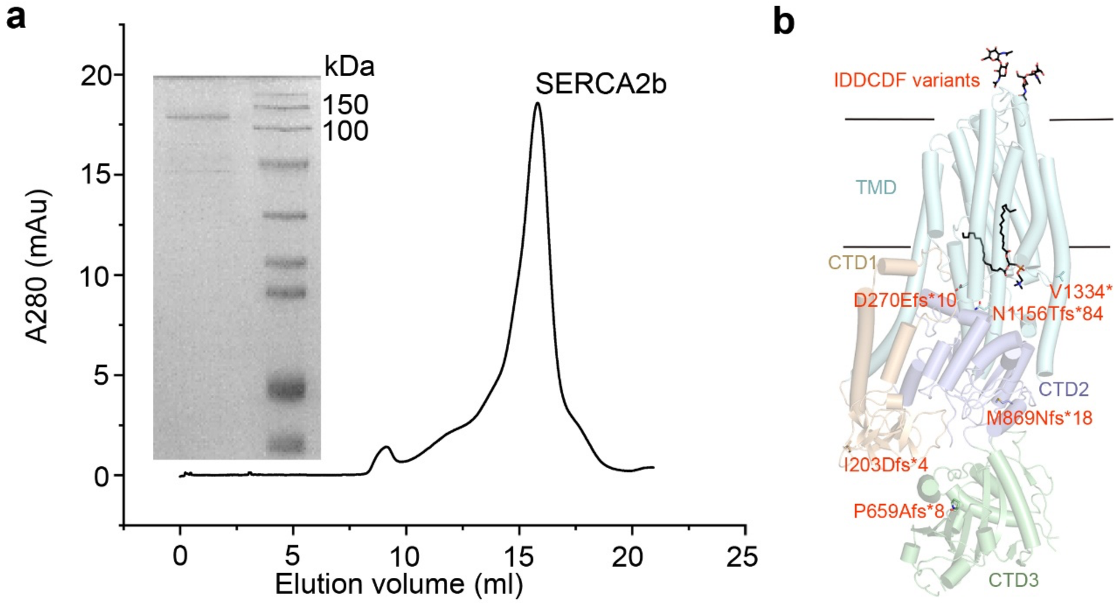

Supplementary Fig. S11 | Protein purification of human SERCA2b protein and structural mapping of pathogenic mutations onto the TMEM94 structure. a, The SERCA2b protein was subjected to SEC. **b,** Six mutants with varying lengths of the cytoplasmic domain deficiency could be reliably mapped to the structure.

Supplementary Table S1: Cryo-EM data collection, refinement and validation statistics

	Condition 1	Condition 2	Condition 3	Condition 4	Condition 5	Condition 6
**Data collection and processing**						
Magnification	96,000	96,000	81,000	81,000	64,000	96,000
Voltage (kV)	300	300	300	300	300	300
Electron exposure ($e^{-}/Å^2$)	50	50	50	50	50	50
Defocus range (μm)	-1.3 to -1.8	-1.3 to -1.8	-1.3 to -1.8	-1.3 to -1.8	-1.3 to -1.8	-1.3 to -1.8
Pixel size (Å)	0.83	0.83	0.8374	0.8374	1.0979	0.83
Symmetry imposed	C1	C1	C1	C1	C1	C1
Movies	3,055	6,129	2,102	4,125	2,073	4,004
Final particle images (no.)	260,789	394,712	170,219	580,228	323,652	318,840
Map resolution (Å)	2.59	2.49	3.24	2.45	2.82	2.92
FSC threshold	0.143	0.143	0.143	0.143	0.143	0.143
Map sharpening *B* factor ($Å^2$)	84.4	81.8	120.6	87.3	107.2	101.6
**Refinement**						
Model composition						
Non-hydrogen atoms	8,358	8,358	8,381	8,401	8,358	8,539
Protein residues	1,048	1,048	1,051	1,054	1,048	1,052
Ligands	5	5	5	5	5	3
B factors ($Å^2$)						
Protein	86.7	100.1	108.3	86.9	129.4	109.4
Ligand	85.9	134.7	120.4	79.0	123.8	80.5
R.m.s. deviations						
Bond lengths (Å)	0.004	0.005	0.007	0.006	0.004	0.004
Bond angles (°)	0.953	0.937	1.297	0.998	0.948	0.977
Validation						
MolProbity score	1.71	1.44	2.00	1.77	1.82	2.25
Clashscore	4.62	4.15	11.46	4.30	6.64	6.87
Poor rotamers (%)	4.06	2.03	1.81	3.61	2.13	5.95
Ramachandran plot						
Favored (%)	98.0	98.0	96.5	97.3	96.7	95.9
Allowed (%)	2.0	2.0	3.5	2.7	3.3	4.1
Outliers (%)	0.0	0.0	0.0	0.0	0.0	0.0

Supplementary References

1 Danko, S. J. & Suzuki, H. The Use of Metal Fluoride Compounds as Phosphate Analogs for Understanding the Structural Mechanism in P-type ATPases. *Methods Mol Biol* **1377**, 195-209 (2016).

2 Wu, M. *et al.* Structure and transport mechanism of the human calcium pump SPCA1. *Cell Res* **33**, 533-545 (2023).

3 Lei, J. & Frank, J. Automated acquisition of cryo-electron micrographs for single particle reconstruction on an FEI Tecnai electron microscope. *J Struct Biol* **150**, 69-80 (2005).

4 Zheng, S. Q. *et al.* MotionCor2: anisotropic correction of beam-induced motion for improved cryo-electron microscopy. *Nat Methods* **14**, 331-332 (2017).

5 Grant, T. & Grigorieff, N. Measuring the optimal exposure for single particle cryo-EM using a 2.6 A reconstruction of rotavirus VP6. *Elife* **4**, e06980 (2015).

6 Zhang, K. Gctf: Real-time CTF determination and correction. *J Struct Biol* **193**, 1-12 (2016).

7 Punjani, A., Rubinstein, J. L., Fleet, D. J. & Brubaker, M. A. cryoSPARC: algorithms for rapid unsupervised cryo-EM structure determination. *Nat Methods* **14**, 290-296 (2017).

8 Rosenthal, P. B. & Henderson, R. Optimal determination of particle orientation, absolute hand, and contrast loss in single-particle electron cryomicroscopy. *J Mol Biol* **333**, 721-745 (2003).

9 Jumper, J. *et al.* Highly accurate protein structure prediction with AlphaFold. *Nature* **596**, 583-589 (2021).

10 Emsley, P. & Cowtan, K. Coot: model-building tools for molecular graphics. *Acta Crystallogr D Biol Crystallogr* **60**, 2126-2132 (2004).

11 Adams, P. D. *et al.* PHENIX: a comprehensive Python-based system for macromolecular structure solution. *Acta Crystallogr D Biol Crystallogr* **66**, 213-221 (2010).
